# The effect of inflammatory diseases associated with opportunistic urogenital infection on the hormonal function of the female reproductive system

**Published:** 2018-11

**Authors:** Abdrakhmanov Azat Rasimovich, Abdrakhmanov Rasim Mindrakhmanovich

**Affiliations:** 1 *Reproductive Health Research Center, Institute of Fundamental Medicine and Biology, Kazan Federal University, Kazan, Russia.*; 2 *University Kazan Clinic, Kazan Federal University, Kazan, Russia.*; 3 *Dermatovenerology Department, Kazan State Medical University, Kazan, Russia.*

**Keywords:** Urogenital infection, Pelvic inflammatory disease, Reproductive system, Estradiol, Infertility

## Abstract

**Background:**

Sexually transmitted infections play a great role in formation of inflammatory diseases of female urogenital system. These diseases can cause a decrease in the level of female sex hormones, as well as the ratio of estrogens and androgens.

**Objective:**

To determine changes in the state of sex hormones in women with inflammatory diseases of ovaries associated with opportunistic urogenital flora.

**Materials and Methods:**

136 women aged 18-39 yr participated in this cross-sectional clinical study. They were examined with the use of a complex of clinical, laboratory and instrumental methods of examination. The patients with confirmed diagnosis of inflammatory disease of reproductive system associated with opportunistic urogenital infection have been under blood analysis to reveal the concentration of follicle-stimulating hormone, luteinizing hormone, and estrogens.

**Results:**

After the treatment the functional capacity of ovary significantly increased, proved by a corresponding increase in the concentration of estradiol hormone in blood serum in all phases of menstrual cycle. According to estradiol in follicular phase its concentration was increased from 421.8±10.8 nmol/L to 581.3±10.2 nmol/L. The elevation in the ratio of estradiol/testosterone hormones in blood serum also is demonstrated.

**Conclusion:**

Inflammatory diseases associated with opportunistic urogenital infection is responsible for a decrease of female sex hormones and the ratio of estrogens and androgens.

## Introduction

The problem of women`s hyperandrogenism and associated pathological conditions significantly increased in recent decades. A growth in clinical manifestations of hyperandrogenism is observed in many countries. With a normal menstrual cycle and the preservation of ovulation processes, hyperandrogenism is accompanied by changes in the function of the skin, hair follicles, and sebaceous glands leading to the formation of vulgar acne, hair loss, and increased hair growth in certain areas of the body. 

However, hormonal examination data gives an important additional information for establishing the correct diagnosis and applying adequate treatment for various inflammatory diseases of the reproductive system.

It is well known that the main hormone responsible for the development of hyperandrogenism is testosterone. The main part of the androgens is produced in peripheral tissues (particularly, in liver, skin, fat, and muscle tissue), in adrenal glands, and in a very small amount, in ovaries, from cholesterol. Modern laboratory technologies allow determining the concentration of free testosterone in the blood. This is considered as the best indicator of androgyny. The study of free testosterone is known as the most accurate analysis showing the active (not chained forms) hormone concentration (1).

Today, more attention is being paid by researchers to the study of relative hyperandrogenism with ovarian diseases, especially inflammatory pathologies associated with sexually transmitted infections (STIs) (2-4). STIs are one of the key reasons for the formation of pelvic inflammatory disease. Inflammatory pathology plays a great role in the formation of dystrophic-sclerotic changes in tissues that lead to functional insufficiency of the affected organs (5-7). 

Previous studies have shown that the presence of a conditionally pathogenic urogenital infection, particularly, mycoplasma infection, causes ovarian failure. It is identified by echogram as hyperechoic zones and takes the second place among the other women`s pelvic organs diseases (11.9%) following metroendometritis (15.9%) (8).

The purpose of this study was to evaluate the serum sexual hormones concentration in the treatment of inflammatory conditions of the female reproductive system.

## Materials and methods

**Study design**

This was a cross-sectional study with an examination of different parameters of the patients with a confirmed diagnosis after the performed treatment. 

**Participants**

A total 572 out of 1280 women aged between 18-39 yr old referred to Reproductive Health Research Center, Kazan, were examined for participating in the study. Finally, 136 women with opportunistic infections and inflammatory diseases of pelvic organs were enrolled. 

**Our inclusion criteria was**

- confirmed etiological and topical diagnosis (i.e. presence of Mycolpasma hominis, Ureaplasma urealyticum, Ureaplasma parvum, Gardnerella vaginalis, Candida spp. in analysis; and the presence of disorders in pelvic organs condition confirmed by instrumental methods of diagnosis);
- Not use of hormonal drugs for past one year;
- Opportunity to participate in the study;
- Informed consent to participate in the study.

The patients managing were performed according to Federal Clinical Recommendations of Russian Scientific Society of Dermatovenerologists and Cosmetologists (9).

The verification of the causative agent of STI was based on the results of laboratory examination using polymerase chain reaction (PCR), and microscopic and microbiological methods of diagnostics. For this purpose the scraping was taken from mucous membrane of reproductive system organs. Collecting of anamnesis, clinical and instrumental methods of examination helped us to examine the condition of women`s pelvic organs. In particular, the fiber-optic video-colposcope "SLV-101" with the possibility of magnification up to 300 times and micrometric capabilities, ultrasonic diagnostic device «Voluson E8 Expert» (made in Japan) (4D/3D scanning, dopplerography, tomographic ultrasound, sonoelastography) with using a transvaginal sensor with an operating frequency of 5MHz were applied.

Myco-ureaplasma infection treatment includes administration of doxycycline monohydrate 100 mg orally 2 times a day with 12 hr intervals for 10 days. Gardnerella infection was treated with metronidazole 500 mg 2 times a day for 7 days. Candidiasis was treated with fluconazole 150 mg orally once. In case of mixed infections-different combination of antibacterial and antiprotozoal medications were applied. Pathogeneses treatment included the administration of vasodilator, enzyme, vitamin, other medications and physiotherapy procedures. The ascertainment of the cure criterion was carried out 4 wk after the end of treatment. Assessment of the functional state of the ovarian system in all patients was determined under standard conditions in 2 main directions: conducting functional test diagnostics and determination of hormones in serum according to conventional methods.

Tests of functional diagnostics were carried out and evaluated according to generally accepted standards strictly on certain days of the menstrual phase, and included:

- Definition of a kariopicnotic index;
- The length of tension of cervical mucus determination;
- Cervix “pupil” symptom;
- Basal temperature measurement during the menstrual cycle.

A kariopicnotic index is defined as the percentage of mature cells with small nuclei to cells having a larger nucleus allowing to indirectly determining the level of androgenization of the female organism

To assess the tension of the cervical mucus, the material was taken with an anatomical tweezers, which were inserted into the cervical canal to a depth of approximately 0.5 cm. Then we sampled the material and evaluated the tension of the mucus with careful spread of the tweezers branches. Cervix “pupil” phenomenon was assessed during the examination using special gynecological mirrors, directing a beam of light onto the outer cervix. Hormonal examinations consisted in determining the concentration of hormones in blood serum also strictly on certain days of each phase of the menstrual cycle. For the objectivity of the study results and the elimination of the external factors influence, the patients were given conditions before donating blood, which included: the exclusion of food intake, alcohol, certain medications that can affect the result (anabolics, contraceptives, etc.), some types of examinations (X-ray, ultrasound, etc.). Blood samples were collected from all participants in the morning and in fasting condition. 

For a correct clinical evaluation of changes in hormonal secretion it was necessary to simultaneously determine the level of the target gland hormone (in this case - the ovarian system) and its regulatory factor (hypothalamic-pituitary system). Therefore, we studied the hormonal profile, which includes:

- Hormonal substances concentrations of the pituitary gland, the most significant of which are: follicle-stimulating hormone (FSH) and luteinizing hormone (LH);
- The level of hormones production by sexual glands-estrogens (estrone–(E1), estradiol–(E2), estriol–(E3)), the main one of which is E2.

Taking into the account that the level of production of female sex hormones depends on the phase of the menstrual cycle, blood sample was taken for analysis three times during the menstrual cycle in accordance with the phase of the menstrual cycle: the follicular phase is 7-8, the ovulatory phase is 13-14, the luteal phase is 21 -22 days of the menstrual cycle.

**Ethical consideration**

The study protocol was approved by Local Ethics Committee of Kazan State Medical University, Kazan, Russia (No 4, 3 February 2015). All participants gave informed consent to participate and publish the details of the study.

**Statistical analysis**

Statistical analysis of data was carried out using SAS® software version 9.3 (SAS inc. Cary, North Carolina, USA), including the statistical database to be evaluated, including all changes, additions, and the receipt and calculation of the variables recorded in this study.

## Results

The effectiveness of etiological treatment is presented in table I. Mycoplasma infection was revealed in 45 cases (33.1%). Ureaplasma urealyticum was examined in 28 patients (20.5%). Ureaplasma parvum was revealed in 23 cases (17.0%), Gardnerella vaginalis was detected in 17 patients (12.5%), Candida spp. - in 13 patients (9.6%). 10 patients had mixed infections (7.3%). After the treatment, mixed infection was detected in 5 cases (3.7%), Ureaplasma urealyticum was revealed in 2 cases (1.5%), Gardnerella vaginalis-in 2 cases (1.5%), and in 1 case-Candida spp. It is clearly seen that the effectiveness of etiological treatment composed 92.3% (p<0.05).

Determination of functional diagnostic tests showed the results presented in table II. For instance, the kariopicnotic index (the percentage of mature cells with small nuclei to cells having a larger nucleus allowing to indirectly determine the level of androgenization of the female organism) before treatment in the follicular phase was 42.1±1.2%, in the ovulatory phase-95.3±1.1%, in luteal-65.3±1.8%. After the treatment these indications were 40.3±0.8%, 92.3±1.2% and 62.3±1.5%, respectively (p<0.05).

The dynamics of the main hormones concentration is demonstrated in Table III (p<0.05). Analysis of the number of hormones in the blood serum before and after treatment showed that the concentration of the main hormone of the female reproductive system (E2) with inflammatory diseases of the ovaries was reduced, but still remained within the reference values.The concentration of E2 before treatment in the follicular phase was 421.8±10.8 nmol/L, in the ovulatory phase-1121.3±10.8 nmol/L, in luteal phase-561.3±11.3 nmol/L. After treatment, the concentration of E2 significantly increased: in the follicular phase up to 581.3±10.2 nmol/L, in the ovulatory phase - up to 1872.6±21.8 nmol/L, in luteal phase - up to 681.3±8.4 nmol/L.

The concentration of free testosterone in the blood serum before and after treatment remained almost unchanged, while the concentration of E2 (the main estrogen) increased significantly in all phases of the menstrual cycle.

The ratio of estradiol/testosterone hormones in blood serum of the examined patients after the treatment significantly increased. Before the treatment in follicular phase it was 150/1, after the treatment it already composed 207/1. In ovulatory phase before the treatment it was 400/1, after treatment it raised up to 668/1, in luteal phase increased from 207/1 to 235/1 within the treatment.

**Table I:** The structure of STIs before and after treatment

**Type of STIs**	**Number of examined patients**	
	**Before treatment**	**After treatment**
Mycoplasma hominis	45 (33.1)	- (0)
Ureaplasma urealyticum	28 (20.5)	2 (1.5)
Ureaplasma parvum	23 (17.0)	- (0)
Gardnerella vaginalis	17 (12.5)	2 (1.5)
Candida speciales	13 (9.3)	1 (0)
Mixed infections	10 (7.3)	5 (3.7)
Total	136 (100)	10 (7.3)

Data presented as n (%).

STIs: Sexually transmitted infections

**Table II:** Dynamics of tests of the functional state of the female reproductive system before and after treatment (mean±SD, p<0.05)

**Index**	**Phases of the menstrual cycle**					
	**Follicular**		**Ovulatory**		**Luteal**	
	**Before **	**After **	**Before **	**After **	**Before **	**After **
Kariopicnotic index (%)	42.1 ± 1.2	40.3 ± 0.8	95.3 ± 1.1	92.3 ± 1.2	65.3 ± 1.8	62.3 ± 1.5
Length of tension of cervical mucus (cm)	1.0 ± 0.3	2.0 ± 0.8	5.3 ± 0.6	6.5 ± 0.5	-	0.8 ± 0.1
Cervix “pupil” symptom (cm)	0.1 ± 0.03	0.2 ± 0.05	0.3 ± 0.04	0.3 ± 0.08	0.1 ± 0.02	0.1 ± 0.04
Basal temperature (°C)	36.5 ± 0.2	36.6 ± 0.3	36.8 ± 0.2	37.0 ± 0.1	36.7 ± 0.2	36.9 ± 0.3

Data presented as n (mean±SD, p<0.05)

**Table III:** Dynamics of the main hormones concentration before and after treatment

**Hormones**	**Reference Values**	**Hormones concentration**	
		**Before treatment**	**After treatment**
FSH (mU/L) Follicular phase	2.8-11.3	8.3 ± 0.8	7.6 ± 0.6
Ovulatory phase	5.8 - 21	16.2 ± 0.6	14.8 ± 1.3
Luteal phase	1.2-9	6.3 ± 0.7	5.2 ± 0.6
LH (mU/L) Follicular phase	2-14	5.8 ± 0.4	7.2 ± 0.6
Ovulatory phase	2-14	78.3 ± 0.6	91.3 ± 0.5
Luteal phase	2-17	9.2 ± 0.1	12.4 ± 0.2
E2 (nmol/L) Follicular phase	77.07-921	421.8 ± 10.8	581.3 ± 10.2
Ovulatory phase	140-2382	1121.3 ± 10.8	1872 ± 21.8
Luteal phase	77.07-1145	561.3 ± 11.3	681.3 ± 8.4
Free testosterone (nmol/L)	0.31-3.78		
Follicular phase		2.8 ± 0.8	2.9 ± 0.2
Ovulatory phase		2.8 ± 0.6	2.8 ± 0.8
Luteal phase		2.8 ± 0.6	2.9 ± 0.1

Data presented as n (mean±SD, p<0.05)

FSH: Follicle-stimulating hormone

LH: Luteinizing hormone

E2: Estradiol

## Discussion

Anamnesis and results of medical examination made it possible to make a preliminary assessment of the function of the female reproductive system. Timeliness of menarche and, normal development of secondary sexual characteristics allow considering estrogenic function as sufficient in the past. The cyclicity of menstruation testifies to the preservation of ovulation, the adequate production of gonadotropins and female sex hormones and, to a certain extent, the intactness of the genital tract.

Going back to the kariopicnotic index (the percentage of mature cells with small nuclei to cells having a larger nucleus allowing to indirectly determine the level of androgenization of the female organism), cytological evaluation of changes in the cellular composition of the vaginal epithelium showed its decrease under the influence of estradiol increased concentration. In addition, the results of functional testing before and after treatment showed that the treatment significantly improved the next indicators: 1) the length of cervical mucus tension showing cyclical changes in the quality of mucus depending on the level of estrogens; 2) the "pupil" symptom of cervix based on cyclic changes in the cervical tone under the influence of estrogens; 3) the basal temperature (change in basal temperature depending on the concentration of sex hormones).

Among three types of estrogens only the concentration of E2 was determined, because, firstly, it is synthesized mainly in the ovaries and, secondly, it is the most active estrogen. E1 synthesized in the liver is a weak form of estrogens. E3 is significantly inferior to estradiol by activity and its role in the organism of a non-pregnant woman is low. Thus, E1 and E3 are not indicative in assessing the functional function of the ovaries.

A permanent concentration of testosterone in all phases of the menstrual cycle is understandable, because women`s testosterone is mainly synthesized by adrenal glands, in peripheral tissues and in a very small amount in ovaries. Accordingly, the pathology of ovaries does not significantly affect to its production. The concentration of FSH decreased after the treatment in all phases of menstrual cycle, with a clear "negative feedback" - an increase in the blood concentration of E2 as a result of the treatment results in a decrease concentration of FSH. With an increase in the concentration of estradiol, the concentration of luteinizing hormone also increases - a clear "positive feedback" is observed.

Franciyanc and colleagues investigated the level of sex hormones, prolactin and sex-steroids-binding globulin in the tissue of adenomyosis and uterine fibroids in the independent and combined development of pathologies. There are violations in the local hormonal background, both with independent adenomyosis, and with uterine myoma (10). Moreover, some investigators examined HIV-infected patients. According to their work the literature data and their findings indicate a significant decrease in the level of sex hormones - estradiol and testosterone-in HIV-infected patients, what shows the necessity for further investigations in this area (11, 12). 

Ishutina and others evaluated the role of cholesterol and estradiol in the development of placental insufficiency in patients with cytomegalovirus infection (CMV) during gestation. These indicators were studied in peripheral blood in 35 pregnant women who underwent reactivation of chronic CMVI (IgG antibody titer to CMV 1: 1600). It has been established that the reactivation of chronic CMV in the third trimester of gestation is accompanied by a decrease in the cholesterol concentration by 23% (p <0.001), and estradiol by 65% (p <0.001) as compared to that of healthy women. Thus, the results obtained in the study allow establishing the important role of violations of the content of total cholesterol and the key steroid hormone of pregnancy (estradiol) in the pathogenesis of placental insufficiency in CMVI during gestation (13). 

However, on the contrary, there are no studies investigating the level of hormonal status in patients with pelvic inflammatory diseases, especially caused by opportunistic urogenital infections. It should be also noted that one of the main causes of impaired fertility are infectious diseases of the reproductive system organs (14). Moreover, male and female infertility associated with various inflammatory diseases of the pelvic organs shows a tendency to its constant growth. The main factor in the continued increase of infertility proportion is delayed and/or irrational treatment of STIs. Thus, infections of the male and female genitourinary system pose a threat to reproductive disorders (15-17). 

Early detection of STIs can prevent not only its further spread and transmission to the offspring, but also reduce the risk of developing serious complications that violate reproductive function. The clinical and social importance of STIs lies precisely in the development of complications, because a long asymptomatic course creates favorable conditions for the spread of infection from the lower sections of the urogenital tract to the pelvic organs performing basic reproductive function of the body (18).

In this study the major number of cases was associated with mycoplasma and ureaplasma infections. This data certifies the previous investigations and the fact that such opportunistic urogenital infection as mycoplasma and ureaplasma can significantly affect the female reproductive system by inducing inflammatory diseases in women pelvic organs. Inflammatory process, especially chronic, in its turn is one of the reasons provoking elaboration of connective tissue in target affected organs leading to their functional failure (19).

## Conclusion

After the treatment the functional capacity of ovary significantly increased, proved by a corresponding increase in the concentration of its main hormone in blood serum. The results of the study show that it is important to know not only the amount of male sex hormones contained in female body, but also the ratio of all hormonal substances-in female body. This requires complex assessment of the state of the reproductive system of patients with relative hyperandrogenism manifestations.
